# TEACHING THE INTERSECTION OF AGING AND DISABILITY FOR FIRST-YEAR STUDENTS

**DOI:** 10.1093/geroni/igac059.406

**Published:** 2022-12-20

**Authors:** Kristine Mulhorn

**Affiliations:** Drexel University, Wallingford, Pennsylvania, United States

## Abstract

The honors college at Drexel included their Freshmen Seminar in a recent effort to offer various courses on the theme of aging across the campus. Usually, the themed courses are for juniors and seniors who can choose electives. This year, the theme of aging was interwoven into two freshmen seminars with a maximum of 20 students. The purpose of this course was to examine the complexity of these two concepts and recognize possible avenues for study, research, and careers based these perspectives. Disability is not only a way of describing how someone lives with biological or physical difference, but disability can be a social identity, and a group recognized in recent civil rights legislation. Aging is not only at the individual level, but an ongoing demographic change that is happening throughout the world. Students reflected on online quizzes on ableism and ageism to gain insights about their own biases and the biases within their chosen career fields. Through class discussions and reflections, students achieved course objectives: 1) describe how society can better prepare individuals to fully participate in society throughout life; 2) recognize key factors affecting those with disability and aging with disability as they navigate education, health services, housing and work; 3) synthesize literature on aging and disability to make recommendations for improving social participation for those either aging with disability or aging into disability; and 4) describe public policy challenges for those over 50 and those who identify as disabled.

